# Supplementary material for: The 3-Year Effect of the Mediterranean Diet Intervention on Inflammatory Biomarkers Related to Cardiovascular Disease
Source: Biomedicines. 2021 Jul 22;9(8):862. doi: 10.3390/biomedicines9080862 (PMC8389558; doi:10.3390/biomedicines9080862)
Supplement: Supplementary file 1 [file biomedicines-09-00862-s001.zip › biomedicines-1240195-supplementary.pdf]

Supplementary Material

The 3-Year Effect of the Mediterranean Diet Intervention on Inflammatory Biomarkers Related to Cardiovascular Disease

Detailed and Expanded Methods

Participants and Study Design

The Predimed study is a parallel-group, single-blind, multicenter, randomized, controlled 5-year clinical trial aimed to assess the effects of the Mediterranean diet (MD) on the primary prevention of cardiovascular diseases (www.predimed.es, accessed on 26 April 2021) [1,2].

Recruitment took place from October 2003 to January 2009 and 7447 high-risk participants were randomly assigned to one of three interventions: a MD supplemented with extra-virgin olive oil (MD-EVOO), a MD supplemented with nuts (MD-Nuts), or a control low-fat diet (LFD). Randomization was performed centrally by means of a computer-generated random-number sequence.

The participants were men (55 to 80 years of age) and women (60 to 80 years of age) without cardiovascular disease at enrollment, who had either type 2 diabetes mellitus or at least three of the following major risk factors: smoking, hypertension (blood pressure ≥140/90 mm Hg or treatment with antihypertensive drugs), LDL-cholesterol concentrations ≥160 mg/dL (or treatment with hypolipidemic drugs), HDL-cholesterol ≤ 40 mg/dL, body mass index (BMI) ≥25 kg/m^2 , or a family history of early-onset coronary heart disease (CHD). All participants provided written informed consent to participate in the study. The design, methodology and eligibility criteria for the PREDIMED study have been described previously [1–3].

In the present study, we selected 285 participants consecutively admitted from primary care centers affiliated to the Hospital Clínic of Barcelona and the University of Valencia.

Diets, Physical Activity and Clinical Measurements

The randomized participants had an annual face-to-face interview with the dietitian and a group session every 3 months. The individual motivational interview included: a 137-item validated food frequency questionnaire (FFQ), a 14-item questionnaire assessing adherence to the MD [4], the Minnesota leisure-time physical activity questionnaire for men [5] and women [6], a 47-item questionnaire about education, lifestyle, history of illnesses and medication use and personal individual recommendations for changes to be introduced in the participant's diet in order to achieve a personalized goal [2,3]. The focus for those participants assigned to both MD groups was shifted in order to increase the intake of vegetables (≥2 servings/d), fresh fruit (≥3 servings/d), legumes, nuts, fish or seafood (≥3 servings/wk), and the use of olive oil for cooking and dressings as previously described, [1,2] while those in the control group were advised to follow a LFD according to the American Heart Association (AHA) guidelines [1,2]. Each intervention group met on separate groupal sessions. In these sessions, participants were provided with descriptions of seasonal foods, shopping lists, weekly meal plans and cooking recipes. The participants in the two MD groups received either EVOO (approximately 1 liter per week for the whole family) or 30 g of mixed nuts per day (15 g of walnuts, 7.5 g of hazelnuts, and 7.5 g of almonds). Supplementary foods were donated, including EVOO (by Hojiblanca and Patrimonio Comunal Olivarero, both in Spain), walnuts (by the California Walnut Commission), almonds (by Borges, in Spain), and hazelnuts (by La Morella Nuts, in Spain). None of the sponsors had any role in the trial design, data analysis, or reporting of the results. Those in the control group received small nonfood gifts. No total calorie restriction was advised, nor was physical activity promoted. The fatty acid composition of the EVOO and nuts used in the trial has been described elsewhere [1–3].

Trained personnel measured body weight and height using calibrated scales and a wall-mounted stadiometer, respectively. Waist circumference was determined midway between the lowest rib and the iliac crest using an anthropometric tape, and blood pressure was measured in triplicate with a validated semiautomatic oscillometer (Omron HEM-705CP) [2,3]. Energy and nutrient intake estimates were obtained from Spanish food composition tables [3]. All these procedures were repeated after 3 years of intervention.

To determine compliance and adherence to intervention with EVOO and nuts, in a random subsample of participants, we measured urinary 3-hydroxytyrosol (3-HT) (the main phenolic compound in EVOO) and plasma proportion of α-linolenic acid (a fatty acid characteristic of walnuts) at baseline and at 3 years, as described previously [1–3].

Supplementary Figures

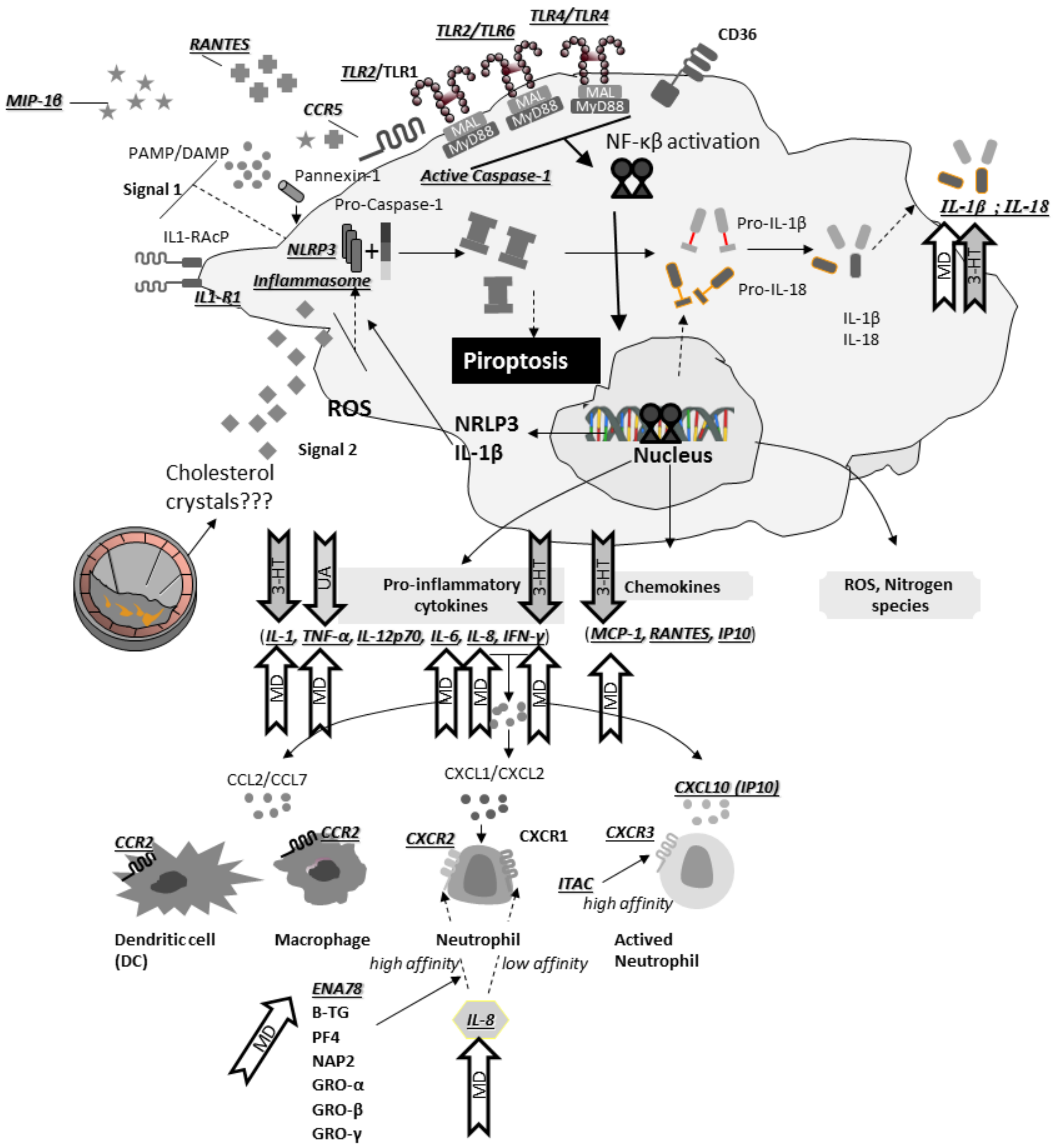

Figure S1. Scheme of plasma inflammatory molecules and genes implied in the different pathways of macrophage activation resulting from chronic inflammation (scheme adapted from references [7–9]). Arrows with labeled "MD" show the molecules affected in vivo after 3 years of MD intervention in comparison to a control low fat diet (LFD).

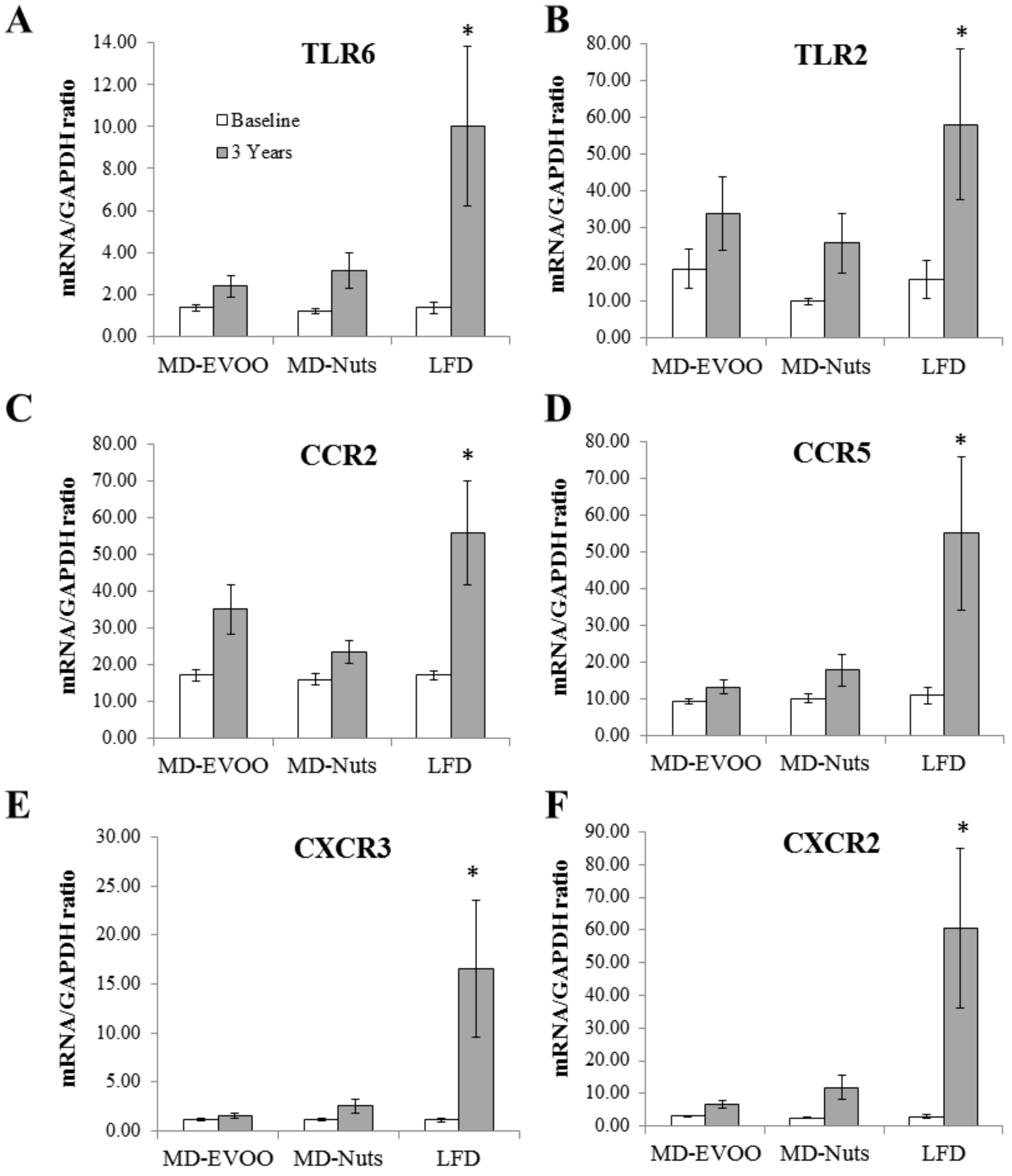

Figure S2. mRNA expression ratios (mean and SEM) of (**A**), TLR6, (**B**), TLR2, (**C**), CCR2, (**D**), CCR5, (**E**), CXCR3 and (**F**), CXCR2 in a pilot study with participants of the Predimed study at baseline and after 3 years of intervention with MD-EVOO (n = 12), MD-Nuts (n = 12) and control LFD (n = 11). * Different from baseline, $p < 0.05$ (Bonferroni post hoc test). These results were not adjusted for covariates. There were no significant changes for CASP, NLRP3, IL1R1 and TLR4.

Supplementary Tables

Table S1. Anthropometric, cardiovascular risk factors and physical activity at baseline and after 3 years of intervention with MD-VOO, MD-Nuts and LFD in patients at high risk for cardiovascular disease.

		MD-EVOO (n = 93)	MD-Nuts (n = 92)	LFD (n = 100)	Repeated-Measures ANOVA [3] Time × Treatment
**Weight (kg)**	Baseline [1]	77.2 ± 13.0	75.3 ± 12.4	76.9 ± 11.8	0.18
	3 years [1]	76.2 ± 13.6 *	75.4 ± 12.7	76.8 ± 12.7	
	Mean 3-year changes[2]	−1.1 (−2.0, −0.1)	0.1 (−0.8, 1.1)	−0.2 (−1.1, 0.7)	
**BMI (kg/m^2)**	Baseline	30.9 ± 4.1	29.8 ± 4.0	30.6 ± 4.3	0.17
	3 years	30.4 ± 4.4 *	29.9 ± 4.3	30.6 ± 4.7	
	Mean 3-year changes	−0.4 (−0.8, −0.05)	0.07 (−0.3, 0.4)	−0.06 (−0.4, 0.3)	
**Waist circumference (cm)**	Baseline	104 ± 10.9	101 ± 10.2	102 ± 10.9	0.81
	3 years	103 ± 11.3	101 ± 10.7	101 ± 13.0	
	Mean 3-year changes	−0.9 (−2.4, 0.5)	−0.3 (−1.8, 1.2)	−0.9 (−2.3, 0.6)	
**Systolic blood pressure (mmHg)**	Baseline	153 ± 16.6	153 ± 16.9	148 ± 18.8	0.26
	3 years	149 ± 17.1	145 ± 16.2 *	145 ± 18.3	
	Mean 3-year changes	−3.7 (−8.1, 0.8)	−7.7 (−12.1, −3.3)	−3.0 (−7.0, 1.0)	
**Diastolic blood pressure (mmHg)**	Baseline	84.3 ± 9.6	86.1 ± 9.0	84.2 ± 10.0	0.49
	3 years	80.0 ± 10.0 *	80.2 ± 8.4 *	79.7 ± 9.1 *	
	Mean 3-year changes	−4.3 (−6.4, −2.2)	−5.9 (−8.0, −3.9)	−4.6 (−6.5, −2.7)	
**Glucose (mg/dL)**	Baseline	141 ± 58.7	127 ± 53.4	128 ± 49.6	0.10
	3 years	129 ± 41.3 *	132 ± 44.0	125 ± 37.3	
	Mean 3-year changes	−11.6 (−22.0, −1.3)	5.0 (−6.2, 16.0)	−3.4 (−12.8, 6.0)	
**Glycated hemoglobin (mg/dL)**	Baseline	6.2 ± 1.8	5.8 ± 1.7	5.8 ± 1.6	0.79
	3 years	6.2 ± 1.3	6.0 ± 1.3	5.8 ± 1.2	
	Mean 3-year changes	0.1 (−0.2, 0.3)	0.2 (−0.1, 0.5)	0.04 (−0.2, 0.3)	
**Triglycerides (mg/dL)**	Baseline	131 ± 70.2	142 ± 88.0	144 ± 70.1	0.77
	3 years	112 ± 40.4 *	124 ± 49.0 *	119 ± 44.0 *	
	Mean 3-year changes	−18.1 (−34.0, −2.4)	−18.0 (−34.7, −1.2)	−24.6 (−38.7, −10.6)	
**Total-cholesterol (mg/dL)**	Baseline	218 ± 38.8	225 ± 37.7	217 ± 35.7	0.59
	3 years	198 ± 34.8 *	213 ± 41.2 *	201 ± 36.7 *	
	Mean 3-year changes	−20.0 (−29.5, −10.2)	−12.4 (-23.0, −1.8)	−16.5 (−25.5, −7.5)	
**HDL-Cholesterol (mg/dL)**	Baseline	54.9 ± 14.0	54.9 ± 12.7	53.9 ± 12.8	0.96
	3 years	52.5 ± 18.0	52.3 ± 14	51.1 ± 13.2 *	

	Mean 3-year changes	−2.3 (−5.0, 0.3)	−2.6 (−5.5, 0.4)	−2.9 (−5.4, −0.3)	
**LDL-Cholesterol (mg/dL)**	Baseline	137 ± 28.8	145 ± 32.2	133 ± 28.5	0.18
	3 years	121 ± 28.0 *	135 ± 29.7 *	128 ± 33.9	
	Mean 3-year changes	−15.6 (−23.8, −7.4)	−9.8 (−19.0, −0.6)	−5.1 (−12.6, 2.5)	
**Cholesterol: HDL-cholesterol ratio**	Baseline	4.1± 1.0	4.2 ± 0.9	4.1 ± 1.1	0.37
	3 years	4.0 ± 0.9	4.3 ± 0.9	4.2 ± 1.0	
	Mean 3-year changes	−0.1 (−0.4, 0.1)	0.1 (−0.2, 0.4)	0.04 (−0.2, 0.3)	
**Physical Activity (MET·min/d)**	Baseline	229 ± 227	200 ± 195	283 ± 295	0.46
	3 years	236 ± 208	245 ± 188 †	291 ± 272	
	Mean 3-year changes	7.6 (−40.2, 55.3)	45.3 (−2.7, 93.3)	8.6 (−37.4, 54.6)	

[1] Values are mean ± SD. [2] Mean differences values at 3 years and at baseline (95% CI). Data analyzed by ANCOVA ($p < 0.05$). [3] Data analyzed by repeated-measures 2-factor ANOVA ($p < 0.05$). Means in a row with superscript without a common letter differ, $p < 0.05$ (Bonferroni post hoc test). Different from baseline, * $p < 0.05$, † p = 0.06–0.08. (Bonferroni post hoc test). LFD, low-fat diet; MD-EVOO, Mediterranean diet supplemented with extra-virgin olive oil; MD-Nuts, Mediterranean diet supplemented with nuts. BMI, body mass index.

Table S2. Consumption of food items and 14-point Mediterranean Diet score at baseline and after 3 years of intervention with MD-VOO, MD-Nuts and LFD in patients at high risk for cardiovascular disease.

		**MD-EVOO** (*n* = 93)	**MD-Nuts** (*n* = 92)	**LFD** (*n* = 100)	**Repeated-Measures ANOVA [3] Time × Treatment**
**EVOO (g/d)**	Baseline[1]	17.1 ± 20.1	10.9 ± 15.7	13.0 ± 17.6	<0.001
	3 years[1]	49.5 ± 8.9 [*,a]	14.2 ± 18.9 [b]	17.1 ± 20.0 [b]	
	Mean 3-year changes [2]	32.4 (27.9, 36.9) [a]	3.3 (−1.3, 7.9) [b]	4.1 (-0.3, 8.5) [b]	
**Refined OO (g/d)**	Baseline	20.4 ± 18.3	24.7 ± 19.0	23.4 ± 18.4	<0.001
	3 years	1.1 ± 7.3 [*,a]	24.8 ± 20.8 [b]	22.3 ± 20.6 [b]	
	Mean 3-year changes	−19.3 (−24.1, −14.5)[a]	0.1 (−4.7, 5.0) [b]	−1.1 (−5.7, 3.6) [b]	
**Total nuts (g/d)**	Baseline	10.5 ± 13.0	13.9 ± 15.3	12.5 ± 15.6	<0.001
	3 years	5.5 ± 6.2 [b]	38.7 ± 34.8 [*,a]	5.7 ± 8.5 [*,b]	
	Mean 3-year changes	−5.0 (−10.1, 0.3)[b]	24.8 (19.6, 30.0) [a]	−6.8 (−11.8, −1.8) [b]	
**Vegetables (g/d)**	Baseline	418 ± 176	376 ± 175	424 ± 195	0.012
	3 years	418 ± 178	425 ± 151 *	388 ± 183	
	Mean 3-year changes	−0.2 (−40.7, 40.4) [ab]	49.5 (8.7, 90.2) [a]	−36.3 (−75.3, 2.8) [b]	
**Legumes (g/d)**	Baseline	21.0 ± 11.0	19.4 ± 7.6	19.4 ± 9.8	0.020
	3 years	25.2 ± 9.2 [*,ab]	28.2 ± 19.4 [*,a]	21.9 ± 10.8 [*,b]	
	Mean 3-year changes	4.2 (1.0, 7.5) [a,b]	8.7 (5.5, 12.0) [a]	2.5 (−0.6, 5.6) [b]	
**Fruits (g/d)**	Baseline	469 ± 221	430 ± 232	446 ± 253	0.88
	3 years	437 ± 198	412 ± 171	410 ± 188	
	Mean 3-year changes	−31.6 (−83.6, 20.3)	−17.7 (−69.9, 34.6)	−35.8 (-85.9, 14.3)	
**Cereals (g/d)**	Baseline	252 ± 128	258 ± 93.5	255 ± 92.5	0.88
	3 years	200 ± 79.1 *	204 ± 76.0 *	194 ± 90.2 *	
	Mean 3-year changes	−52.4 (−77.2, −27.6)	−54.0 (−78.9, −29.0)	−60.7 (−84.6, −36.8)	
**Fish or seafood (g/d)**	Baseline	102 ± 43.0	102 ± 48.1	104 ± 52.8	0.74
	3 years	117 ± 49.8 *	118 ± 54.8 *	115 ± 51.0	
	Mean 3-year changes	15.1 (3.6, 26.7)	16.6 (5.0, 28.2)	10.6 (−0.6, 21.7)	
**Meat or meat products (g/d)**	Baseline	147 ± 62.7	150 ± 51.8	143 ± 55.7	0.30
	3 years	128 ± 50.0 [*,ab]	135 ± 45.0 [*,a]	114 ± 52.4 [*,b]	
	Mean 3-year changes	−18.8 (−31.6, −6.1)	−15.7 (−28.5, −2.8)	−29.0 (−41.2, −16.7)	
**Pastries, cakes or sweets (g/d)**	Baseline	19.3 ± 23.6	23.4 ± 37.0	22.0 ± 35.0	0.92
	3 years	13.9 ± 17.0	16.5 ± 18.5 *	17.0 ± 21.3	
	Mean 3-year changes	−5.4 (−12.2, 1.4)	−6.8 (−13.7, −0.06)	−5.0 (−11.6, 1.5)	
**Dairy products (g/d)**	Baseline	379 ± 185	354 ± 198	413 ± 280	0.34
	3 years	416 ± 235	355 ± 189	404 ± 257	

	Mean 3-year changes	37.2 (−9.1, 83.6)	1.3 (−45.3, 47.9)	−9.3 (−54.0, 35.3)	
**Alcohol (g/d)**	Baseline	6.8 ± 13.0	9.2 ± 21.2	8.7 ± 15.3	0.47
	3 years	7.7 ± 13.0	7.7 ± 13.0	8.8 ± 12.4	
	Mean 3-year changes	0.9 (−1.9, 3.7)	−1.6 (−4.4, 1.3)	0.06 (−2.7, 2.8)	
**Wine (mL/d)**	Baseline	46.0 ± 94.6	52.1 ± 96.4	55.4 ± 109	0.73
	3 years	62.5 ± 116	62.6 ± 116	63.3 ± 97.1	
	Mean 3-year changes	16.5 (0.8, 32.3)	10.5 (-5.3, 26.4)	7.8 (−7.3, 23.0)	
**MD Score**	Baseline	4.7 ± 4.3	5.4 ± 4.2	6.1 ± 4.5	<0.001
	3 years	9.9 ± 1.8 [*,a]	10.7 ± 1.8 [*,a]	8.8 ± 2.3 [*,b]	
	Mean 3-year changes	5.1 (4.2, 6.1) [a]	5.3 (4.4, 6.3) [a]	2.7 (1.8, 3.6) [b]	

[1] Values are mean ± SD. [2] Mean differences values at 3 years and at baseline (95% CI). Data analyzed by ANCOVA ($p < 0.05$). [3] Data analyzed by repeated-measures 2-factor ANOVA ($p < 0.05$). Means in a row with different superscript letters are significantly different, $p < 0.05$ (Bonferroni post hoc test). *Different from baseline, $p < 0.05$ (Bonferroni post hoc test). [†] Difference with p = 0.06–0.08. LFD, low-fat diet; MD-EVOO, Mediterranean diet supplemented with extra-virgin olive oil; MD-Nuts, Mediterranean diet supplemented with nuts.

Table S3. Energy and nutrient data at baseline and after 3 years of intervention with MD-VOO, MD-Nuts and LFD in patients at high risk for cardiovascular disease.

		MD-EVOO (n = 93)	MD-Nuts (n = 92)	LFD (n = 100)	Repeated-Measures ANOVA [3] Time × Treatment
**Energy (kcal/d)**	Baseline[1]	2389±643	2397±581	2364 ± 620	0.037
	3 years[1]	2150±395 [*,ab]	2292±381 [a]	2043 ± 432 [*,b]	
	Mean 3-year changes [2]	−240 (-358, −121) [ab]	−105 (−225, 14.5) [a]	−321 (−436, −207) [b]	
**Protein (% E)**	Baseline	16.9 ± 2.8	16.7 ± 2.5	17.2 ± 2.7	<0.001
	3 years	16.6 ± 5.1 [ab]	17.5 ± 5.0 [a]	15.4 ± 3.9 [*,b]	
	Mean 3-year changes	−0.3 (−1.1, 0.5) [b]	0.7 (−0.1, 1.6) [b]	−1.8 (−2.6, −1.0) [a]	
**Carbohydrate (% E)**	Baseline	43.0 ± 7.0	42.9 ± 6.0	43.0 ± 7.0	0.75
	3 years	37.4 ± 14.7 *	38.3 ± 11.0 *	36.9 ± 10.3 *	
	Mean 3-year changes	−5.6 (−8.4, −2.8)	−4.6 (−7.4, −1.8)	−6.1 (−8.8, −3.4)	
**Fiber (g/d)**	Baseline	28.3 ± 7.8	28.5 ± 9.2	27.8 ± 8.5	0.002
	3 years	27.4 ± 6.4 [a]	29.9 ± 7.4 [a]	24.2 ± 7.6 [*,b]	
	Mean 3-year changes	−0.9 (−2.8, 1.1) [ab]	1.3 (−0.6, 3.3) [a]	−3.6 (−5.5, −1.7) [b]	
**Total fat (% E)**	Baseline	38.3 ± 6.3	37.9 ± 5.4	37.5 ± 6.2	<0.001
	3 years	39.1 ± 11.3 [a]	42.0 ± 11.5 [*,a]	34.5 ± 9.1 [*,b]	
	Mean 3-year changes	0.9 (−1.6, 3.3) [a†]	4.1 (1.6, 6.6) [a]	−3.1 (−5.4, −0.7) [b]	
**SFA (% E)**	Baseline	10.0 ± 2.3	10.0 ± 2.0	9.8 ± 2.2	0.18
	3 years	9.5 ± 3.2	9.5 ± 2.8	8.6 ± 2.4 *	
	Mean 3-year changes	−0.5 (−1.1, 0.2)	−0.5 (−1.1, 0.2)	−1.2 (−1.8, −0.6)	
**MUFA (% E)**	Baseline	18.8 ± 4.1	18.1 ± 3.4	18.3 ± 3.9	0.001
	3 years	20.9 ± 6.2 [*, a]	20.7 ± 5.6 [*,a]	17.5 ± 5.1 [b]	
	Mean 3-year changes	2.1 (0.8, 3.4) [a]	2.5 (1.2, 3.8) [a]	−0.8 (−2.1, 0.5) [b]	
**PUFA (% E)**	Baseline	6.1 ± 2.0	6.7 ± 2.4	6.3 ± 2.0	<0.001
	3 years	5.6 ± 1.8 [b]	8.4 ± 3.7 [*,a]	5.4 ± 2.4 [*,b]	
	Mean 3-year changes	−0.5 (−1.3, 0.2) [b]	1.8 (1.1, 2.5) [a]	−1.0 (−1.6, −0.3) [b]	
**Linoleic acid (g/d)**	Baseline	13.7 ± 6.8	14.8 ± 6.5	14.0 ± 6.3	<0.001
	3 years	11.1 ± 3.0 [*,b]	17.9 ± 8.5 [*,a]	11.0 ± 4.5 [*,b]	
	Mean 3-year changes	−2.5 (−4.2, −-0.8) [b]	3.1 (1.4, 4.8) [a]	−3.0 (−4.6, −1.3) [b]	
**α- linolenic acid (g/d)**	Baseline	1.5 ± 0.7	1.6 ± 0.8	1.6 ± 0.8	<0.001
	3 years	1.3 ± 0.4 [*,b]	2.2 ± 1.1 [*, a]	1.1 ± 0.4 [*,b]	
	Mean 3-year changes	−0.2 (−0.4, −0.003) [b]	0.6 (0.4, 0.8) [a]	−0.4 (−0.6, −0.2) [b]	
**Marine n-3 fatty acids (g/d)**	Baseline	0.83 ± 0.45	0.77 ± 0.44	0.84 ± 0.50	0.20
	3 years	0.96 ± 0.44 *	0.94 ± 0.48 *	0.88 ± 0.51	
	Mean 3-year changes	0.14 (0.03, 0.24)	0.17 (0.06, 0.28)	0.04 (−0.07, 0.14)	

**Cholesterol (mg/d)**	Baseline	387 ± 121	384 ± 108	394 ± 172	0.16
	3 years	363 ± 124	382 ± 109	350 ± 105 *	
	Mean 3-year changes	−23.5 (−53.7, 6.8)	−2.8 (−33.1, 27.6)	−43.6 (−72.7, −14.4)	

[1] Values are mean ± SD. [2] Mean differences in values at 3 years and at baseline (95% CI). Data analyzed by ANCOVA ($p < 0.05$). [3] Data analyzed by repeated-measures 2-factor ANOVA ($p < 0.05$). Means in a row with different superscript letters are significantly different, $p < 0.05$ (Bonferroni post hoc test). * Different from baseline, $p < 0.05$ (Bonferroni post hoc test). † Difference with p = 0.06–0.08. LFD, low-fat diet; MD-EVOO, Mediterranean diet supplemented with extra-virgin olive oil; MD-Nuts, Mediterranean diet supplemented with nuts.

Table S4. Baseline and 3 year mean changes of biomarkers of adherence to supplemental foods by intervention group.

	MD-EVOO Group	MD-Nuts Group	LFD Group	p [2]
Urinary 3-hydroxytyrosol, µg/L†				
Baseline	137.3 (90.8–183.8)	152.8 (107.8–198.7)	148.2 (103.2–193.2)	0.89
Mean 3-year changes	+278.1 (115.5–440.7) [*,a]	-25.7 (-183.0–131.6) [b]	−49.8 (−207.0–107.5) [b]	0.008
Plasma α-linolenic acid, % ‡[1]				
Baseline	0.32 (0.27–0.36)	0.29 (0.24–0.34)	0.34 (0.30–0.39)	0.26
Mean 3-year changes	−0.01 (−0.06–0.05) [a]	+0.11 (0.05–0.16) [*,b]	−0.05 (−0.10–0.00) [a]	<0.001

[1] Data are expressed as mean (95% confidence interval). [2] Obtained by ANOVA. Means in a row with different superscript letters are significantly different, $p < 0.05$ (Bonferroni post-hoc test). * Changes for which the 95% confidence interval does not include zero are significantly different from the baseline. † Performed in 91 participants (29/31/31, respectively). ‡ Performed in 105 participants (33/33/39, respectively). MD, Mediterranean diet; EVOO, extra-virgin olive oil; LFD, low-fat diet.

Table S5. Concentrations of plasma circulating inflammatory chemokines, cytokines and CRP at baseline and after 3 years of intervention with MD-EVOO, MD-Nuts and control LFD in participants of the Predimed trial.

					p Value of Repeated-Measures ANOVA	*p* Value of ANCOVA for Differences		
		**MD-EVOO (*n* = 93)**	**MD-Nuts (*n* = 92)**	**LFD (*n* = 100)**	**Time × treatment**	**MD-EVOO vs. LFD**	**MD-VOO vs. MD-Nuts**	**MD-Nuts vs. LFD**
MCP-1, pg/mL		*n* = 91	*n* = 92	*n* = 97				
	Baseline[1]	3.71 (3.31–4.16)	3.09 (2.71–3.41)	3.05 (2.73-3.41)				
	3y	3.04 (2.71–3.41) [b, **]	2.48 (2.21–2.78) [a,**]	2.96 (2.65–3.31) [ab]	0.020	0.067	1.00	0.035
MIP-1β, pg/mL		*n* = 92	*n* = 92	*n* = 100				
	Baseline	8.67 (7.83–9.59)	8.92 (8.06–9.88)	7.75 (7.03–8.54)				
	3y	8.09 (7.34–8.92) *	8.04 (7.29–8.87) **	7.38 (6.72–8.11)	0.46	1.00	1.00	0.64
RANTES, pg/mL		*n* = 93	*n* = 92	*n* = 100				
	Baseline	821.5 8690.4–977.5)	835.1 (699.9–996.6)	820.7 (693.4–971.5)				
	3y	661.6 (567.4–771.4) *	571.3 (488.7–667.8) **	625.7 (539.1–726.2) *	0.54	1.00	0.82	1.00
IP-10, pg/mL		*n* = 93	*n* = 92	*n* = 100				
	Baseline	32.4 (26.8– 39.2) [b]	41.7 (34.3 – 50.5) [a]	26.7 (22.2–32.1) [b]				
	3y	29.1 (24.7 – 34.2) [ab]	37.5 (31.8 – 44.2) [a]	28.4 (24.2–33.2) [b]	0.20	0.34	1.00	0.39
ENA78, pg/mL[3]		*n* = 85	*n* = 87	*n* = 94				
	Baseline	130.0 (104.2–162.1)	95.2 (76.5–118.4)	111.9 ± 2.8				
	3y	79.5 (62.9–100.6) [ab,**]	64.5 (51.1–81.4) [b,**]	99.7 (79.1–124.7) [a]	0.024	0.063	1.00	0.21
I-TAC, pg/mL[3]		n = 85	*n* = 87	*n* = 93				
	Baseline	3.66 (2.97–4.47)	4.35 (3.55–5.27)	3.57 (2.92–4.33)				
	3y	3.34 (2.70–4.10)	3.74 (3.04–4.57)	5.31 (3.42–5.02)	0.12	0.35	1.00	0.15
IL-1β, pg/mL		*n* = 90	*n* = 89	*n* = 97				
	Baseline	0.18 (0.14–0.21)	0.16 (0.13–0.20)	0.12 (0.08–0.15)				
	3y	0.14 (0.11–0.17) *	0.13 (0.09–0.16) *	0.14 (0.11–0.18) †	0.005	0.015	1.00	0.014
IL-6, pg/mL		*n* = 91	*n* = 88	*n* = 99				
	Baseline	0.98 (0.80–1.18)	0.93 (0.74–1.13)	0.69 (0.54–0.86)				
	3y	0.77 (0.61–0.93) *	0.75 (0.60–0.92) *	0.78 (0.63–0.94)	0.006	0.010	1.00	0.033
IL-8, pg/mL		*n* = 90	*n* = 91	*n* = 99				
	Baseline	1.52 (1.29–1.78)	1.33 (1.13–1.57)	1.19 (1.02–1.39)				
	3y	1.26 (1.07–1.49) *	1.07 (0.91–1.26) *	1.33 (1.14–1.56)	0.017	0.029	1.00	0.014
IL-12p70, pg/mL		*n* = 91	*n* = 89	*n* = 100				
	Baseline	1.40 (1.10–1.73)	1.53 (1.22–1.90)	1.12 (0.87 – 1.41)				

	3y	1.21 (0.95–1.51)	1.24 (0.97–1.55) *	1.22 (0.97–1.50)	0.065	0.28	1.00	0.08
IL-18, pg/mL		n = 92	n = 92	n = 100				
	Baseline	7.16 (6.35–8.08)	7.75 (6.86–8.75)	7.10 (6.32–7.98)				
	3y	7.37 (6.60–8.23)	7.88 (7.05–8.81)	7.06 (6.34–7.85)	0.86	1.00	1.00	1.00
TNF-α, pg/mL		n = 90	n = 89	n = 99				
	Baseline	2.32 (1.88–2.84)	2.06 (1.64–2.54)	1.67 (1.32–2.06)				
	3y	1.85 (1.47–2.29) *	1.52 (1.18–1.91) *	1.91 (1.54–2.33)	0.006	0.047	1.00	0.021
IFN-γ, pg/mL		n = 91	n = 89	n = 99				
	Baseline	10.28 (8.33–12.64)	8.38 (6.72–10.39)	7.83 (6.35–9.61)				
	3y	8.04 (6.37–10.08) *	5.84 (4.55–7.42) *	8.41 (6.73–10.46)	0.035	0.18	1.00	0.042
CRP, mg/dL		n = 35	n = 24	n = 42				
	Baseline	0.31 (0.22–0.44)	0.27 (0.18–0.41)	0.33 (0.24–0.45)				
	3y	0.20 (0.15–0.27) *	0.16 (0.11–0.23) *	0.24 (0.18–0.32) †	0.73	1.00	1.00	1.00

[1] Data are expressed as mean (95% confidence interval). Means in a row with different superscript letters are significantly different, $p < 0.05$ (Bonferroni post hoc test). * Different from baseline, * $p < 0.05$; ** $p \leq 0.001$; † $p = 0.05$–0.08. Statistical analysis was done using repeated-measures 2-factor ANOVA or ANCOVA for between group differences. Both adjusted for age, gender, energy intake, BMI, smoking status, physical activity and drug use. MCP-1 indicates monocyte chemoattractant protein-1; MIP, macrophage inflammatory protein; RANTES, regulated on activation normal T cell expressed and secreted, IP, interferon gamma-induced protein; ENA, epithelial neutrophil-activating protein; I-TAC, inducible T-cell alpha chemoattractant; IL, interleukin; TNF, tumor necrosis factor; IFN, interferon; and CRP, C-reactive protein.

Table S6. Pearson's correlation of 3-year changes in cytokines in the three groups in the Predimed study.

3-Year Changes	TNF-α	IL-1β	IL-6	IL-8
		**MD-EVOO group**		
**IFN-γ**	0.829 **	0.684 **	0.770 **	0.735 **
**TNF-α**		0.891 **	0.827 **	0.892 **
**IL-1β**			0.744 **	0.817 **
**IL-6**				0.842 **
		**MD-Nuts group**		
**IFN-γ**	0.774 **	0.622 **	0.690**	0.801 **
**TNF-α**		0.801 **	0.738**	0.820 **
**IL-1β**			0.701**	0.702 **
**IL-6**				0.764 **
		**LFD group**		
**IFN-γ**	0.853 **	0.770 **	0.801 **	0.814 **
**TNF-α**		0.889 **	0.749 **	0.834 **
**IL-1β**			0.656 **	0.736 **
**IL-6**				0.749 **

MD, Mediterranean diet; EVOO, extra-virgin olive oil; LFD, low-fat diet. ** $p < 0.001$.

References

1. Estruch, R.; Ros, E.; Salas-Salvadó, J.; Covas, M.-I.; Corella, D.; Arós, F.; Gómez-Gracia, E.; Ruiz-Gutiérrez, V.; Fiol, M.; Lapetra, J.; et al. Retraction and Republication: Primary Prevention of Cardiovascular Disease with a Mediterranean Diet. *N. Engl. J. Med.* **2018**, *368*, 2441–2442, doi:10.1056/NEJMc1806491.
2. Estruch, R.; Martinez-Gonzalez, M.A.; Corella, D.; Salas-Salvadó, J.; Ruiz-Gutiérrez, V.; Covas, M.I.; Fiol, M.; Gómez-Gracia, E.; López-Sabater, M.C.; Vinyoles, E.; et al. Effects of a Mediterranean-Style Diet on Cardiovascular Risk Factors: A randomized trial. *Ann. Intern. Med.* **2006**, *145*, 1–11, doi:10.7326/0003-4819-145-1-200607040-00004.
3. Martinez-Gonzalez, M.A.; Corella, D.; Salas-Salvadó, J.; Ros, E.; Covas, M.I.; Fiol, M.; Wärnberg, J.; Arós, F.; Ruíz-Gutiérrez, V.; Lamuela-Raventos, R.M.; et al. Cohort Profile: Design and methods of the PREDIMED study. *Int. J. Epidemiology* **2010**, *41*, 377–385, doi:10.1093/ije/dyq250.
4. Schröder, H.; Fitó, M.; Estruch, R.; Martínez-González, M.A.; Corella, D.; Salas-Salvadó, J.; Lamuela-Raventós, R.; Ros, E.; Salaverría, I.; Fiol, M.; et al. A Short Screener Is Valid for Assessing Mediterranean Diet Adherence among Older Spanish Men and Women. *J. Nutr.* **2011**, *141*, 1140–1145, doi:10.3945/jn.110.135566.
5. Elosua, R.; Marrugat, J.; Molina, L.; Pons, S.; Pujol, E. Validation of the Minnesota Leisure Time Physical Activity Questionnaire in Spanish Men. *Am. J. Epidemiology* **1994**, *139*, 1197–1209, doi:10.1093/oxfordjournals.aje.a116966.
6. Elosua, R.; Garcia, M.; Aguilar, A.; Molina, L.; I Covas, M.; Marrugat, J. Validation of the Minnesota Leisure Time Physical Activity Questionnaire in Spanish Women. *Med. Sci. Sports Exerc.* **2000**, *32*, 1431–1437, doi:10.1097/00005768-200008000-00011.
7. Suárez-Rivero, J.; Pastor-Maldonado, C.; Povea-Cabello, S.; Álvarez-Córdoba, M.; Villalón-García, I.; Talaverón-Rey, M.; Suárez-Carrillo, A.; Munuera-Cabeza, M.; Sánchez-Alcázar, J. From Mitochondria to Atherosclerosis: The Inflammation Path. *Biomed.* **2021**, *9*, 258, doi:10.3390/biomedicines9030258.
8. Wolf, D.; Ley, K. Immunity and Inflammation in Atherosclerosis. *Circ. Res.* **2019**, *124*, 315–327, doi:10.1161/circresaha.118.313591.
9. Noels, H.; Weber, C.; Koenen, R.R. Chemokines as Therapeutic Targets in Cardiovascular Disease. *Arter. Thromb. Vasc. Biol.* **2019**, *39*, 583–592, doi:10.1161/atvbaha.118.312037.
